# Jian Gan powder ameliorates immunological liver injury in mice by modulating the gut microbiota and metabolic profiles

**DOI:** 10.1186/s40001-024-01827-2

**Published:** 2024-04-20

**Authors:** Kun Li, Yadong Cui, Xue Zheng, Chunyan Min, Jian Zhang, Zhanpeng Yan, Yu Ji, Fei Ge, Hualiang Ji, Fangshi Zhu

**Affiliations:** 1 Affiliated Hospital of Integrated Traditional Chinese and Western Medicine, Nanjing University of Chinese Medicine, 100 Hongshan Road, Building 9, Nanjing, 210046 Jiangsu People’s Republic of China; 2 Department of Gastroenterology, Hai’an Hospital of Traditional Chinese Medicine Affiliated to Medical College of Yangzhou University, Nantong, People’s Republic of China; 3 Jiangsu Province Academy of Traditional Chinese Medicine, Nanjing, People’s Republic of China; 4 College of Pharmaceutical Science, Soochow University, Suzhou, People’s Republic of China; 5 Department of Gastroenterology, Affiliated Haian People’s Hospital of Nantong University, 17 Zhong Ba Zhong Road, Hai’an, 226600 Jiangsu People’s Republic of China; 6 Suzhou Institute for Drug Control, Suzhou, People’s Republic of China

**Keywords:** Fecal metabolomics, Gut microbiota, Immunological liver injury, Jian Gan powder, 16S rRNA gene sequencing

## Abstract

**Background:**

Immunological liver injury (ILI) is a common liver disease associated with the microbiota-gut-liver axis. Jian Gan powder (JGP) exhibits both protective and therapeutic effects on hepatitis virus-induced ILI in the clinic. However, the underlying mechanisms remain elusive. The aim of this study is to investigate the hepatoprotective effects and associated mechanisms of JGP in the context of gut microbiota, utilizing a mouse model of ILI.

**Methods:**

The mouse model was established employing Bacillus Calmette-Guérin (BCG) plus lipopolysaccharide (LPS). Following treatment with JGP (7.5, 15, or 30 g/kg), serum, liver, and fresh fecal samples were analyzed. 16S rRNA gene sequencing and untargeted metabolomics profiling were performed to assess the role of JGP on the gut microbiota and its metabolites.

**Results:**

JGP treatment markedly reduced serum IFN-γ, IL-6, IL-22, and hepatic p-STAT3 (phosphorylated transducer and activator of transcription-3) expression. In contrast, JGP increased the percentage of proliferating cell nuclear antigen-positive liver cells in treated mice. Fecal 16S rRNA gene sequencing revealed that JGP treatment restored the levels of *Alloprevotella*, *Burkholderia-Caballeronia-Paraburkholderia*, *Muribaculum*, *Streptococcus*, and *Stenotrophomonas*. Additionally, metabolomics analysis of fecal samples showed that JGP restored the levels of allylestrenol, eplerenone, phosphatidylethanolamine (PE) (P-20:0/0:0), sphingomyelin (SM) d27:1, soyasapogenol C, chrysin, and soyasaponin I.

**Conclusions:**

JGP intervention improves ILI by restoring gut microbiota and modifying its metabolic profiles. These results provide a novel insight into the mechanism of JGP in treating ILI and the scientific basis to support its clinical application.

**Supplementary Information:**

The online version contains supplementary material available at 10.1186/s40001-024-01827-2.

## Background

The liver plays a crucial role in physiological homeostasis and a range of metabolic processes [1, 2]. The prevalence of liver injury caused by stress insults, alcohol abuse, drug exposure, viral infections, and other risk factors has increased in recent years. The most common liver injury is ILI, which is implicated in the pathogenesis of acute liver failure, chronic hepatitis, and liver fibrosis [3]. Current approaches for ILI treatment include (1) immune modulators, such as interferon [4]; (2) antiviral medications, including lamivudine, adefovir, entecavir, and telbivudine [5, 6]; (3) liver protection by silibinin treatment [7]; and (4) glucocorticoids, such as prednisone [8, 9]. Although many drugs have been used clinically for treating ILI, the use of some medications is limited by resistance, serious side effects, and low therapeutic efficacy [10–13]. Hence, identifying effective therapeutic approaches for ILI is essential [14].

The gut microbiota is a potential therapeutic target for ILI. Changes in the gut flora are strongly correlated with liver diseases [15]. The gut microbiota communicates with the liver through the gut-liver axis. Liver disease reshapes intestinal microbial communities and inhibits the secretion of intestinal and microbial metabolites to the liver, aggravating the disease [16]. Consequently, the proper functioning of the gut and liver relies on maintaining the homeostasis of the gut-liver axis[17–20].

Traditional Chinese medicine (TCM) controls liver disease progression by modulating intestinal microbial communities and metabolic profiles [21–23]. Moreover, TCM demonstrates efficacy in both the prevention and treatment of ILI. [24–27]. JGP contains 11 herbs and is widely used to treat ILI induced by hepatitis B virus infections [28]. We hypothesized that JGP improves ILI by modulating gut microbial structure and metabolic profiles. We successfully established a mouse model of ILI using Bacillus Calmette-Guérin (BCG) plus lipopolysaccharides (LPS). Changes in gut microbial composition were investigated by 16S rRNA gene sequencing. Intestinal metabolites were analyzed using untargeted metabolomics.

## Materials and methods

### Animals

Male BALB/c mice (age: 6–8 weeks) were purchased from the Laboratory Animal Management Department of the Shanghai Family Planning Research Institute (Shanghai, China) and maintained in an environmentally controlled room (22 ± 1 °C, 45% relative humidity, 12-h light/12-h dark cycle). The animals were acclimated for 1 week.

The experimental protocols were established according to the ethical guidelines of the Basel Declaration and were approved by the Animal Ethics Committee of the Jiangsu Province Hospital of Integration of Chinese and Western Medicine (Approval number: AEWC-20220119-185). All experiments were performed in accordance with the ARRIVE guidelines.

### Preparation of JGP suspension

*Panax ginseng* CA Mey (Araliaceae), *Paeonia lactiflora Pall.* (Paeoniaceae), *Astragalus mongholicus Bunge* (Fabaceae), *Salvia miltiorrhiza Bunge* (Lamiaceae), *Curcuma longa L.* (Zingiberaceae), *Cyperus rotundus L.* (Cyperaceae), *Citrus aurantium L.* (Rutaceae), *Sophora flavescens Aiton* (Fabaceae), *Artemisia capillaris Thunb.* (Asteraceae), *Bupleurum falcatum L.* (Apiaceae), *and Glycyrrhiza glabra L.* (Fabaceae) were mixed at a ratio of 2:4:4:2:2:1:1:4:2:1:1. The mixture was soaked and boiled repeatedly with distilled water. Then, the mixture was filtered, and the supernatant was concentrated and sterilized. JGP (1.0 g of raw herb per mL) was obtained and stored at -20℃. The ratio was reset according to the concentration requirements.

### Equipment, chemicals, and reagents

Microplate reader (Infinite M1000 Pro, Tecan); fixed speed vortex mixer (model VXMNFS; Ohaus); Sorvall ST 16R centrifuge (Thermo Fischer Scientific); rotary evaporator (EYELA OBS-2100); enhanced BCA protein assay kit (Lot No. 090120200929; Beyotime Biotechnology); 5% phenol aqueous solution (Lot L02M12G140441; Shanghai Yuanye Bio-Technology Co., Ltd.); D-( +)-glucose (Lot LRAA8593; Sigma-Aldrich); vitriol (AR; Lot 20210902; Soochow Qiangsheng); precision electronic balance (EL303, Mettler Toledo, Shanghai Co., Ltd.; accuracy, 0.0001 g); ultrasonic cleaner (SB-5200DT; power, 240 W; frequency, 40 kHz; Scientz Biotechnology Co. Ltd., Ningbo, China); high-speed refrigerated centrifuge (Eppendorf 5810 R); JGP (Lot 200106; Hai’an Hospital of Traditional Chinese Medicine). Paeoniflorin (Lot AF20070752, purity > 98%) and hesperidin (Lot AF9061522, purity > 98%) were purchased from Chengdu Aifa Biotechnology Co., Ltd. (Chengdu, China). Chromatographic grade acetonitrile and formic acid, deionized or ultrapure water, and analytical grade ethyl alcohol were used in all experiments.

Extracts were analyzed on an Agilent 1290 Infinity II ultra-high performance liquid chromatography (UHPLC) system connected to an Agilent 6538 Accurate-Mass Q-TOF mass spectrometry (MS) system (Agilent Technologies, Inc., Santa Clara, CA, USA). LC/MS data were analyzed using Agilent MassHunter Qualitative Analysis software (version B.05.00, Build 4.0.479.5, Service Pack 3; Agilent Technologies, Inc., Santa Clara, CA, USA).

### Preparation of JGP water extract and reference compounds

JGP (100 g) was soaked in boiling water (500 mL) for 20 min twice. After filtration, 100 mL of the extract was evaporated under vacuum at 60 °C, and water was added to obtain a water extract (12.5 mg/mL). Reference compounds were dissolved in pure methanol, and the concentration of each standard solution was prepared as follows: paeoniflorin, 104 μg/mL; hesperidin, 80 μg/mL. All solutions were sterilized through a 0.22 μm filter.

### UHPLC-MS analysis of JGP water extract

Chromatographic separations were performed on an Acquity UPLC HSS T3 analytical column (2.1 mm × 100 mm; id, 1.8 μm; Waters, USA). The operating conditions were as follows: column temperature, 30 °C; flow rate, 0.3 mL/min; injection volume, 5.0 μL. Elution was carried out using a system consisting of solvent A (0.1% formic acid in water) and solvent B (0.1% formic acid in acetonitrile) and the following elution conditions: 10% B for 2 min, 10% to 95% B for 20 min, 95% B for 25 min, 95% to 10% B for 2 min. Mass spectra were obtained in positive and negative modes using the following source parameters: gas temperature, 350 °C; gas flow rate, 10 L min^−1^; nebulizer gas pressure, 40 psi; Vcap, 3500; fragmentor, 135 V. Reference masses were *m/z* 121.0509 and *m/z* 1221.9906. The results are shown as base peak chromatograms (BPCs) with an *m/z* range of 50–2500.

### Measurement of polysaccharide content

JGP (30 g) was soaked in 150 mL of deionized boiling water for 20 min twice. After filtration, deionized water was added to the extract to a volume of 300 mL. Then, 1 mL of the JGP extract was mixed with 199 mL of deionized water to obtain a concentration of 0.5 mg raw herb/mL. Five milliliters of the extract were mixed with ethanol to a final concentration of 80% (v/v), and the sample was precipitated overnight at 4 °C. The sample was centrifuged at 7995* g* for 10 min at 4 ℃ to obtain JGP polysaccharides. Total polysaccharide concentration was measured at 490 nm using the phenol–sulfuric acid method and was calculated using the linear equation (*y* = 6.8308 *x*-0.0053, *R*^2^ = 0.9995) at a glucose concentration range of 0.005–0.1 mg/mL.

### Determination of protein content

JGP (30 g) was soaked in 150 mL of deionized boiling water for 20 min twice. The extract was filtered, and deionized water was added to a volume of 300 mL. Then, 1 mL of the extract was added to 19 mL of deionized water, and the solution was mixed by vortexing. Protein concentration was measured at 562 nm using the bicinchoninic acid (BCA) protein assay kit according to the manufacturer’s instructions. The detection range was 0.025 – 0.5 mg/mL (*y* = 1.0112 *x* + 0.023, *R*^2^ = 0.9998).

### Induction of ILI and animal grouping

After 1 week of acclimation, mice were randomly divided into six groups of five animals: normal group (NG), model group (MG, induced for ILI), positive control group (PCG, 15 g/kg/day of JGP), low-dose JGP group (JGP-L, 7.5 g/kg/day), medium-dose JGP group (JGP-M, 15 g/kg/day) and high-dose JGP group (JGP-H, 30 g/kg/day). The MG and JGP groups were injected with 2.5 mg/200 μL BCG (Catalog No. 202010, Rebio, China) in the tail vein to induce ILI, and the NG and PCG were injected with the same volume of saline through the tail vein [29, 30]. The PCG and JGP groups were treated with JGP for 12 days by gastric gavage once daily. The NG and MG received the same dose of water. On day 12, the MG and JGP groups were injected with 7.5 μg/200 μL LPS (Catalog No. L6529; Sigma, USA) in the tail vein [31], and the NG and PCG were injected with the same volume of saline through the tail vein.

### Enzyme-linked immunosorbent assay (ELISA)

The serum concentrations of IFN-γ, IL-6, IL-10, and IL-22 were measured using the following ELISA kits according to the manufacturer’s protocols: IFN-γ (Catalog No. ab100689, Abcam), IL-6 (Catalog No. ab222503, Abcam), IL-10 (Catalog No. ab255729, Abcam), and IL-22 (Catalog No. ab223857, Abcam). Briefly, standards, blank controls, and test samples (100 μL/well) were added to 96-well plates. Next, a biotinylated antibody (50 μL/well) was added to each well and incubated at 37 °C for 90 min. Unbound biotinylated antibody was removed using washing buffer (PBS with a mild detergent). Streptavidin–horseradish peroxidase (100 μL/well) was added to the plates, followed by incubation at 37 °C for 30 min. TMB (100 μL/well) was added, followed by incubation at 37 °C for 10 min in the dark. The reaction was interrupted using a stop solution, and optical density was measured at a wavelength of 450 nm using a microplate reader. The concentration of each sample was quantified using a standard curve.

### Western blotting

Hepatocytes were treated with 1 mL of cold Western and IP cell lysate (Catalog No. P0013, Beyotime) and 1% PMSF (Catalog No. ST505, Beyotime) and frozen for 30 min. The lysate was centrifuged at 12,000 rpm for 10 min at 4 °C, and protein concentration in the supernatant was measured using the BCA assay (Catalog No. 23225, Thermo Fisher Scientific) [32]. Three microliters were collected from the solution, and 27 μL of phosphate-buffered saline (PBS) was added (Additional file 3).

In each group, 25 μg of denatured protein was separated by 10% SDS-PAGE (stacking gel voltage, 80 V; running gel voltage, 120 V) and transferred to a 0.45-μm polyvinylidene difluoride membrane for 2 h at a constant current of 300 mA. The membrane was incubated with 5% skim milk powder for 1 h at room temperature under agitation. Then, the membrane was washed with Tris-buffered saline and Tween 20 (TBST) and incubated with the following antibodies at 4 °C under shaking overnight: GAPDH (1:10,000, Catalog No. AP0063, Bioworld), p-STAT3 (1:1000, Catalog No. 9145T, CST), and STAT3 (1:1000, Catalog No. 9139, CST). The membrane was washed with TBST and incubated with goat anti-mouse IgG and goat anti-rabbit IgG (1:3000, Catalog No. A0208, Beyotime) at room temperature for 1 h. Immunoreactive bands were visualized using a chemiluminescence image analysis system (Tanon Technology Co., Shanghai, China) and analyzed using Quantity One version 4.6.2 [33, 34].

### Histological analysis

Liver tissue slices were cut at a thickness of 4 μm, deparaffinized, rehydrated, incubated in 1 × citrate buffer for 5 min, and subjected to high-pressure antigen retrieval. Peroxidase activity was blocked with hydrogen peroxide, and the sections were treated with a PCNA antibody (1:200; Catalog No. ab29, Abcam) overnight at 4 °C. Then, the samples were incubated with a horse anti-mouse Texas Red-conjugated secondary antibody (1:400), stained with diaminobenzidine, and counterstained with hematoxylin. The sections were dehydrated in a graded ethanol series, cleared in xylene, mounted in Permount, and observed under an optical microscope at 200 × magnification [35, 36].

### Flow cytometry

Liver tissues were cut into pieces and digested at 37 °C for 45 min with DMEM containing hyaluronidase (1.5 mg/mL, Sigma–Aldrich, USA), collagenase type 1A (1.5 mg/mL, Sigma–Aldrich, USA), and deoxyribonuclease I (20 U/mL, Sigma–Aldrich, USA). The samples were passed through a 70-μm nylon cell strainer to produce a single cell suspension and resuspended in a cold flow cytometry buffer (1% bovine serum albumin and 0.1% NaN_3_ in PBS). Fc receptors were blocked with a rat anti-mouse CD16/CD32 antibody (BD Pharmingen, USA). Cells were stained with the following fluorochrome-conjugated anti-mouse antibodies: CD45-BV421, CD11b-BV510, F4/80-FITC, and Ki67-APC (all from BioLegend, USA). The number of cells was measured using 7-amino-actinomycin D (eBioscience, USA). Flow cytometry was performed using a Gallios flow cytometer (Beckman, USA), and data were analyzed using Kaluza software version 1.3.

### Fecal DNA extraction and lllumina MiSeq sequencing

Total microbial DNA was extracted from fresh fecal samples using the EZNA Stool DNA Kit (Omega Bio-Tek). PCR primers targeting the variable region of the 16S /ITS2 rDNA gene were designed. After 35 PCR cycles, sequencing adapters, and barcodes were added for amplification. PCR amplification products were separated by agarose gel electrophoresis. DNA was purified using the AxyPrep PCR Clean-up Kit (Axygen) and quantified using the Quant-iT PicoGreen dsDNA Assay Kit (Invitrogen). Libraries were quantified using a QuantiFluor-ST Blue Fluorescence Quantification System (Promega, USA). The pooled library was sequenced on an Illumina platform using a paired-end protocol (2 × 250 bp).

Paired-end reads were assigned to samples based on their unique barcodes, and barcodes and primers were trimmed. Paired-end reads were merged using FLASH version 1.2.8 (for 16S)/PEAR version 0.9.6 (for ITS2). Quality control of raw reads was performed using fqtrim version 0.94. Chimeric sequences were removed using Vsearch version 2.3.4. After dereplication using DADA2, we obtained a feature table and feature sequences. Alpha diversity and beta diversity were analyzed using QIIME2 and R version 3.5.2, and the same number of sequences was extracted randomly by reducing the number of sequences to the minimum of some samples, and relative abundance was used in bacteria taxonomy. Sequence alignment was performed using BLAST, and the alignment database was SILVA and NT-16S.

#### UPLC-Q-TOF/MS-based metabolic profiling

Fecal samples were thawed on ice, and metabolites were extracted with 50% methanol. Metabolites were analyzed by LC‐MS using a high‐resolution tandem mass spectrometer (TripleTOF 5600 Plus, Sciex, UK).

Peak picking, peak grouping, retention time correction, second peak grouping, and annotation of isotopes and adducts were performed using XCMS software. Raw data files were converted to the mzXML format and processed using XCMS, CAMERA, and metaX toolbox in R. Each ion was identified by combining retention time and *m/z* values. Peak intensities were measured, and a three-dimensional matrix containing arbitrarily assigned peak indices (retention time‐*m/z* pairs), sample names (observations), and ion intensities (variables) was generated.

Metabolites were annotated by matching the molecular mass data (*m/z*) of samples to those from the KEGG and HMDB databases. If a mass difference between observed and reference values was less than 10 ppm, the metabolite was annotated, and its molecular formula was identified based on unique isotope patterns. Metabolite identification was validated using an in‐house fragment spectrum library.

Peak intensities were preprocessed using metaX. The features detected in less than 50% of quality control (QC) samples or 80% of biological samples were removed, and values for missing peaks were extrapolated using the k‐nearest neighbor algorithm to improve data quality. Principal component analysis (PCA) of preprocessed data was performed to detect outliers and assess batch effects. Signal intensities were normalized using locally estimated scatterplot smoothing. In addition, relative standard deviations in metabolic features across QC samples were calculated, and those greater than 30% were removed.

P-values were adjusted for multiple tests using the Benjamini and Hochberg false discovery rate procedure. Supervised orthogonal partial least squares discriminant analysis (OPLS‐DA) was conducted using metaX to discriminate between variables. Variable importance in projection (VIP) values were calculated, and relevant features were selected using a VIP cut‐off of 1.0.

### Statistical analysis

Data were expressed as means ± standard errors of the means. Intergroup differences in organ index, protein expression, hematoxylin–eosin (HE) staining, and fecal metabolite concentrations were evaluated by one-way analysis of variance. OTUs with significant differential abundance across groups were identified using Welsh’s *t*-test and linear discriminant analysis (LDA) effect size (LEfSe). A p-value of less than 0.05 was considered statistically significant. The correlation coefficient between fecal metabolites and the gut microbiota at the genus level was calculated by Spearman’s rank correlation, and heatmaps were generated using GraphPad Prism version 8.0.2.

## Results

### Chemical profiling of the JGP extract

**Fig. 1:**
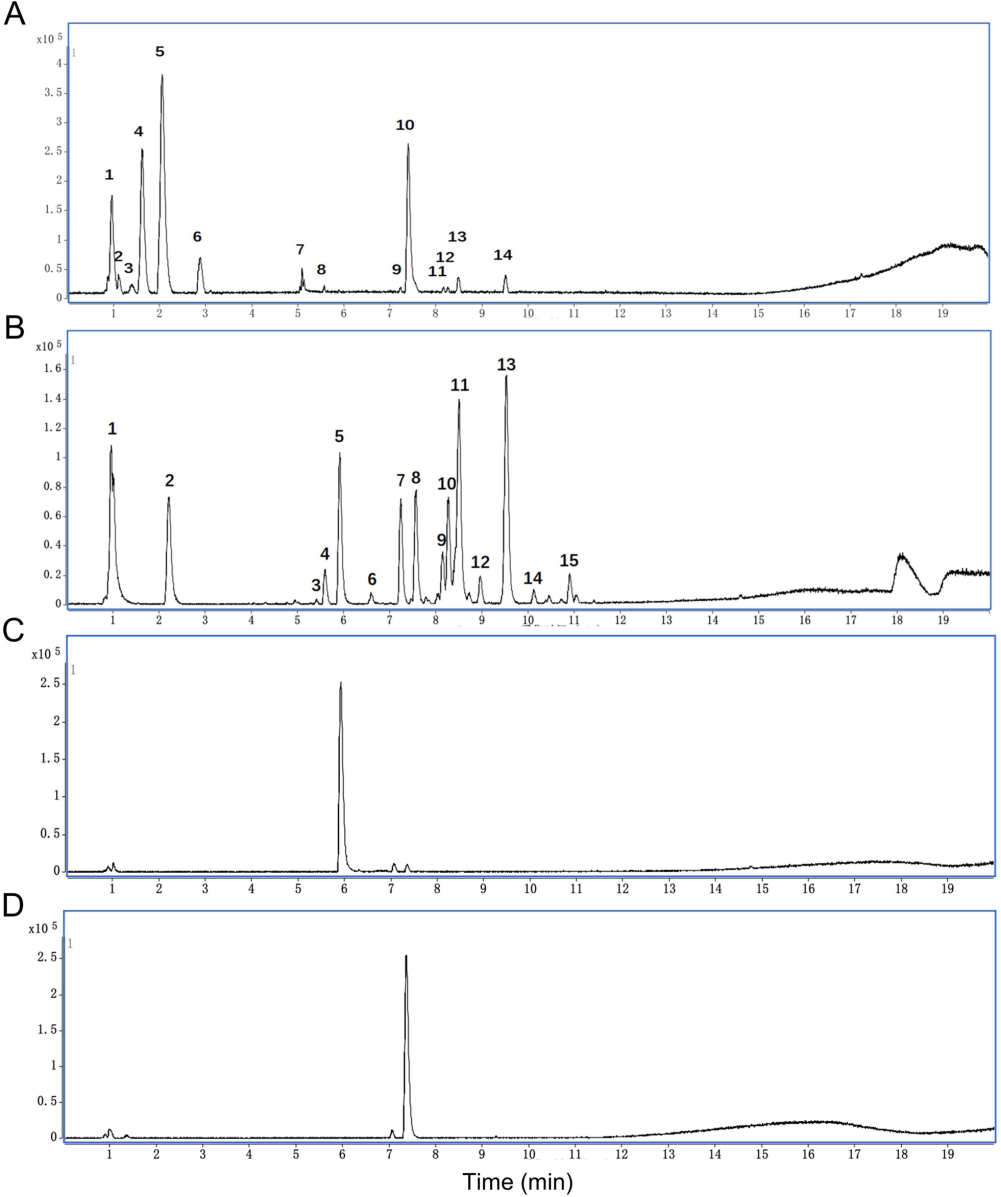
Base peak chromatograms of Jian Gan powder water extract. **A** Positive ion mode, **B** Negative ion mode. **C** Paeoniflorin in the negative ion mode. **D** Hesperidin in the negative ion mode

Twenty-nine well-separated chromatographic peaks in JGP water extract were found in the positive and negative ion modes of BPC **(**Fig. 1A–D**)**. Thirty-five compounds were tentatively identified by their accurate masses relative to reported references (Additional file 2: Tables S1-1, S1-2). Thirty-five compounds were tentatively identified by their accurate masses relative to reported references (Additional file 2: Tables S1-1, S1-2), including peaks 3, 4, 5, and 6 (alkaloids), 8 (monoterpenoid), and 14 (ginsenoside) in positive mode; peaks 1 (quinone), 3, 4, and 5 (monoterpenoids), 6 and 8 (flavonoid glycosides), 7 and 9 (salvianolic acid), 12 and 14 (flavonoids), 13 (ginsenoside), and 15 (glycyrrhizin) in negative mode.

Although small molecules were identified in the extract, polysaccharides and proteins may also be present because these large molecules are extractable by water. The concentrations of carbohydrates and proteins in the extract were determined. Polysaccharide content was determined at 490 nm using the phenol–sulfuric acid method and calculated using a linear equation (*y* = 6.8308 *x*-0.0053, *R*^2^ = 0.9995) at a glucose concentration range of 0.005–0.1 mg/mL. The total polysaccharide content was 12.03 mg/g. Protein concentration was measured at 562 nm using the BCA assay at a concentration range of 0.025–0.5 mg/mL (*y* = 1.0112 *x* + 0.023, *R*^2^ = 0.9998). The total protein content was 60.65 mg/g.

### JGP exhibits anti-inflammatory effect and promotes liver cell proliferation

**Fig. 2:**
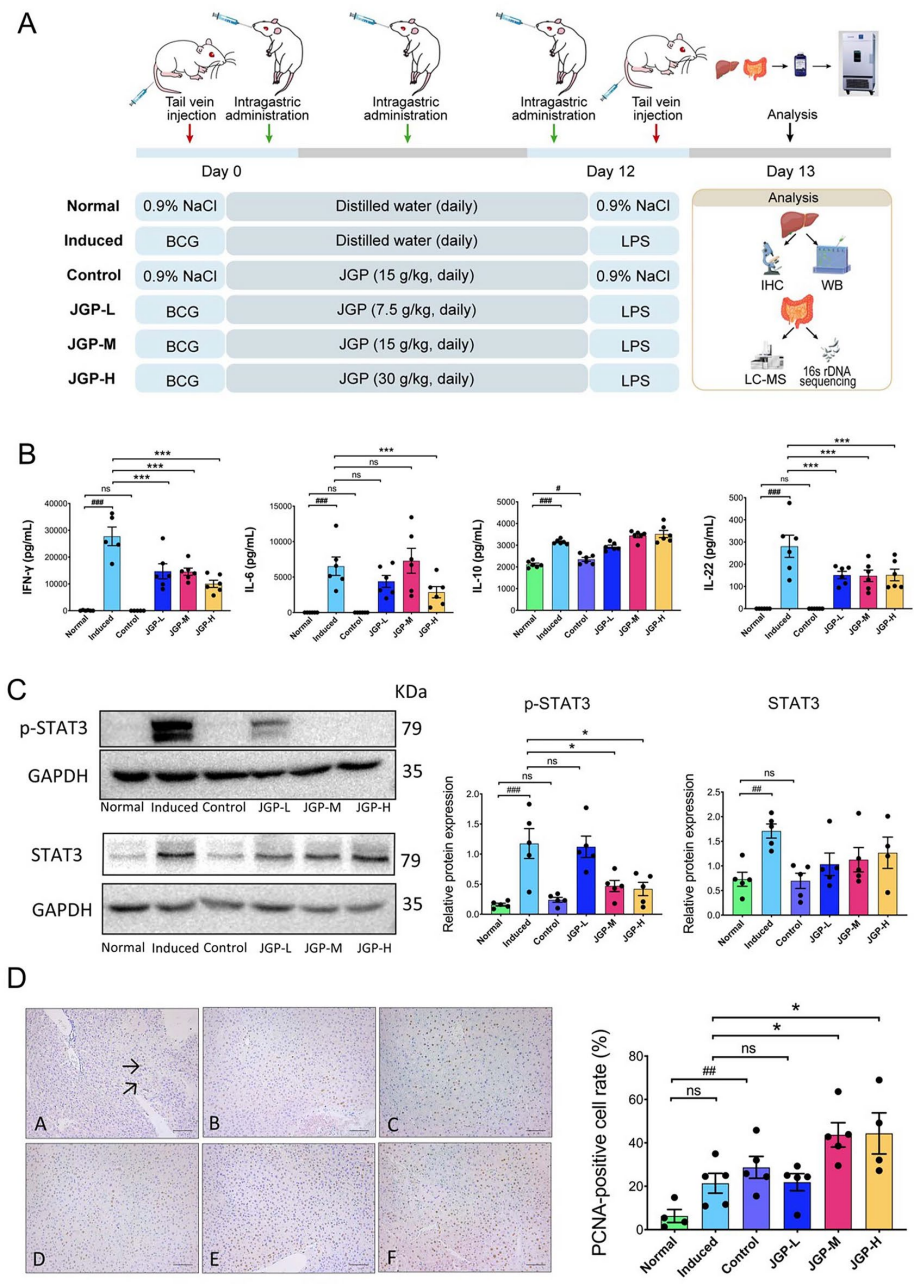
Role of Jian Gan powder (JGP) in BCG + LPS-induced immunological liver injury (ILI) in BALB/c mice. **A** Experimental schedule. **B** ELISA of the serum levels of interferon-gamma (IFN-γ), interleukin (IL)-6, IL-10, and IL-22. **C** Western blotting analysis of STAT3 and p-STAT3 protein levels in the liver of experimental and control groups (five mice per group). **D** Representative images of proliferating cell nuclear antigen (PCNA) staining (× 200) and the percentage of PCNA-positive cells (arrowhead). (**A** normal group; **B** model group [induced for experimental ILI]; **C** positive control group; **D** JGP-L group; E: JGP-M group; F: JGP-H group). Bars: 100 μm. Data were analyzed using one-way analysis of variance and were presented as mean ± SEM. ^#^*p* < 0.05, ^##^*p* < 0.01, ^###^*p* < 0.001; ^*^p < 0.05, ^**^p < 0.01, ^***^p < 0.001

The mouse model of ILI was established successfully, and the protective effect of JGP on the liver was assessed (Fig. 2A). BCG and LPS significantly increased the serum levels of IFN-γ (*p* < 0.001), IL-6 (*p* < 0.001), IL-10 (*p* < 0.001), and IL-22 (*p* < 0.001). All JGP doses reversed the effects of BCG and LPS on IFN-γ (*p* < 0.001) and IL-22 (*p* < 0.001), and JGP-H reversed their effects on IL-6 (*p* < 0.001) (Fig. 2B). The protein expression levels of STAT3 and p-STAT3 in the liver were measured by western blotting. BCG and LPS significantly increased the expression of STAT3 (*p* < 0.01) and p-STAT3 (*p* < 0.001), and JGP-M and JGP-H inhibited p-STAT3 expression (*p* < 0.05) (Fig. 2C). STAT3 expression data were corroborated by HE staining (Additional file 1: Fig. S1A, B).

The effect of JGP on Kupffer cell (KC) infiltration and cellular proliferation in the liver was evaluated. JGP-H significantly decreased the percentage of KCs (*p* < 0.05) in ILI mice (Additional file 1: Fig. S2A), potentially reducing liver damage induced by BCG + LPS. Proliferating cell nuclear antigen (PCNA) is a cell proliferation marker [37, 38]. The percentage of PCNA-positive cells was significantly higher (*p* < 0.01) in the PCG than in the NG. Additionally, JGP-M and JGP-H increased the percentage of PCNA-positive cells (*p* < 0.05) compared with the MG (Fig. 2D). These data were corroborated by flow cytometry findings (Additional file 1: Fig. S3B). These results suggest that JGP has anti-inflammatory properties and promotes cellular proliferation in the liver of BCG + LPS-treated mice.

### JPG modulates the gut microbial structure

**Fig. 3:**
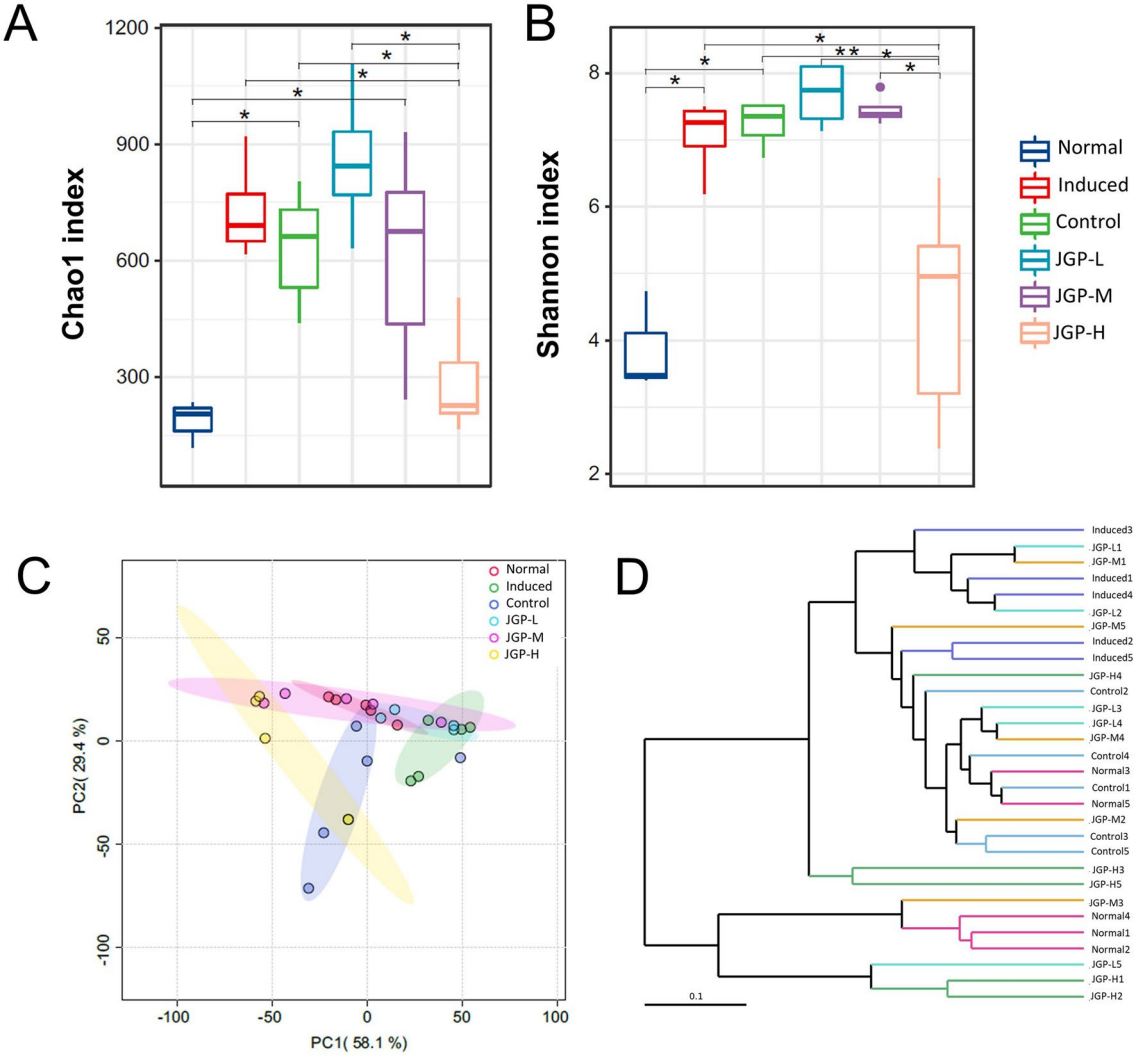
Community richness and diversity in the intestine of the experimental and control groups. **A**, **B** Chao 1 and Shannon indexes were used to evaluate alpha diversity. **C** OTU-based principal component analysis of intestinal microbial communities. **D** Multiple sample similarity tree. Data are mean ± SEM. ^*^p < 0.05, ^**^p < 0.01. PC: principal component

According to the sequencing of bacterial 16S rRNA from fecal samples, the total number of generated tags was 2497,891, with an average of 83,263 tags per sample. Rarefaction curves showed that the sequencing depth was high (Additional file 1: Fig. S3A). Alpha diversity analysis showed that microbial community richness (Chao 1) and diversity (Shannon index) increased significantly in the MG, PCG, JGP-L, and JGP-M groups compared with the NG (*p* < 0.05), and JGP-H reversed this effect (*p* < 0.05) **(**Fig. 3A, B**)**. The number of OTUs was higher in the PCG and MG than in the NG (Additional file 1: Fig. S3B). In turn, the number of OTUs was lower in the JGP-H group than in the MG, indicating that JGP treatment influenced gut microbial diversity in ILI mice. Ordination of Bray–Curtis dissimilarity by principal coordinate analysis (PCoA) revealed the separation of the six groups (Fig. 3C). Furthermore, compared with the MG, bacterial community structure was similar between JGP-treated groups and the NG, suggesting that JGP modulates gut microbial structure. In addition, the sample clustering tree showed significant differences among the six groups, and the level in JGP-treated groups was close to that in the NG (Fig. 3D).

### JGP regulates the gut microbial composition

**Fig. 4:**
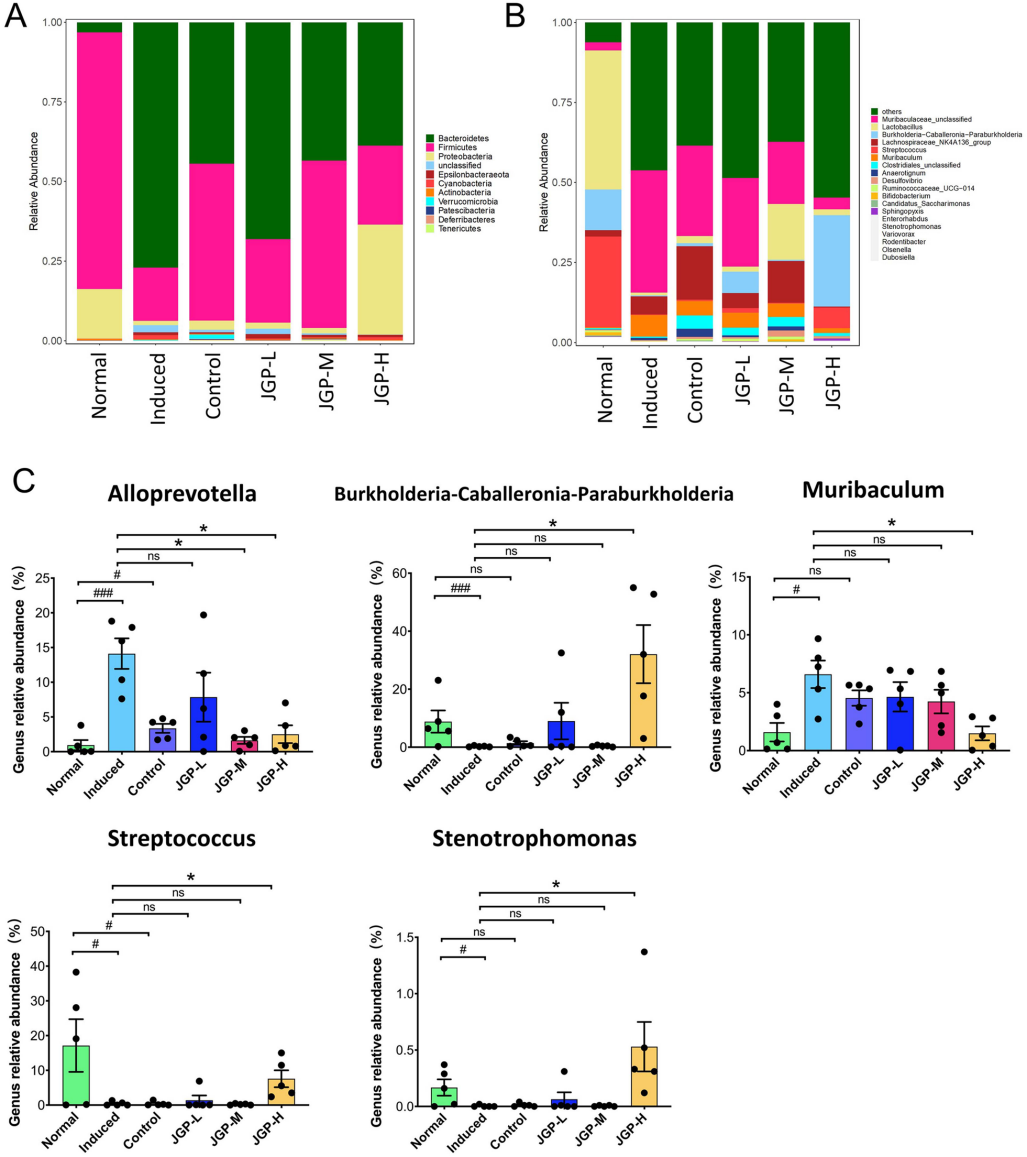
Gut microbial structure in the experimental and control groups. **A** Abundance of intestinal bacteria at the phylum level in the experimental and control groups. **B** Abundance of intestinal bacteria at the genus level. **C** Relative abundance of bacterial genera in the experimental and control groups. Data were analyzed by one-way analysis of variance and presented as mean ± SEM. ^#^*p* < 0.05; ^###^*p* < 0.001; ^*^p < 0.05; ^***^P < 0.001. JGP-L, JGP-M, and JGP-H represent low, intermediate, and high doses of JGP

The microbial species at the phylum and genus levels and relative abundances are shown in Fig. 4. The most abundant phylum in the NG, PCG, and JGP-M group was *Firmicutes*. The dominant phylum in the MG and JGP-L group was *Bacteroidetes*. The most abundant phylum in the JGP-H group was *Proteobacteria* (Fig. 4A). The alterations in the core microbiota at the genus level are displayed in Fig. 4B, C. ILI decreased the relative abundance of *Streptococcus, Variovorax, Lactobacillus, Burkholderia-Caballeronia-Paraburkholderia,* and *Stenotrophomonas* and increased the relative abundance of *Muribaculum* and *Alloprevotella*. JGP treatment reversed the effect of ILI on the relative abundance of *Alloprevotella*, *Burkholderia-Caballeronia-Paraburkholderia*, *Muribaculum*, *Streptococcus*, and *Stenotrophomonas* (*p* < 0.05).

**Fig. 5:**
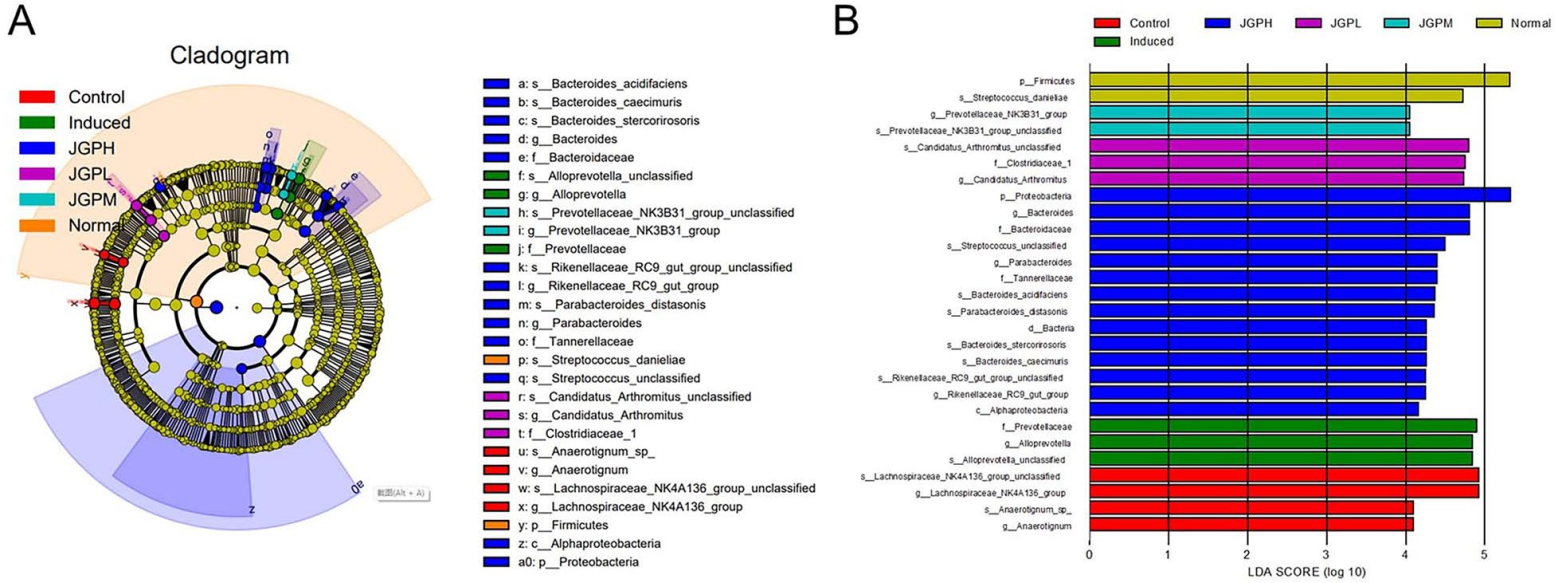
Differentially abundant taxa in fecal samples from the experimental and control groups. **A** Cladogram of bacterial taxa in fecal samples from the experimental and control groups based on linear discriminant analysis (LDA) effect size (LEfSe) analysis. The letters p, c, o, f, and g represent phylum, class, order, family, and genus. **B** LEfSe analysis of differentially abundant (log_10_ LDA scores > 4) bacterial taxa in fecal samples from the experimental and control groups. JGP-L, JGP-M, and JGP-H correspond to a low, intermediate, and high dose of JGP

LEfSe analysis was performed to identify bacterial taxa associated with ILI and JGP treatment. The discriminative features of bacterial taxa were identified using an LDA score of > 4.0 (Fig. 5A, B). Two taxa—*p_Firmicutes* and *s_Streptococcus danieliae*—predominated in the NG. Lachnospiraceae_NK4A136 unclassified, g_Lachnospiraceae_NK4A136, s_Anaerotignum_sp, and g_Anaerotignum predominated in the PCG, while f_Prevotellaceae, g_Alloprevotella and s_Alloprevotella unclassified predominated in the MG. Moreover, s_Candidatus arthromitus unclassified, f__Clostridiaceae_1, and g_Candidatus arthromitus were enriched in the JGP-L group; g_Prevotellaceae_NK3B31 and s_Prevotellaceae_NK3B31 unclassified were enriched in the JGP-M group. Fourteen taxa predominated in the JGP-H group, including p_Proteobacteria, g_Bacteroides, f_Bacteroidaceae, and s_Streptococcus unclassified.

Correlation analyses showed that *Streptococcus* was positively associated with *Lactobacillus* and *Burkholderia-Caballeronia-Paraburkholderia*. *Muribaculaceae unclassified* was positively correlated with *Muribaculum* and *Alloprevotella* and negatively associated with *Streptococcus* and *Burkholderia-Caballeronia-Paraburkholderia* (Additional file 1: Fig. S4A, B).

### Effect of JGP on the fecal metabolic profile of ILI mice

**Fig. 6:**
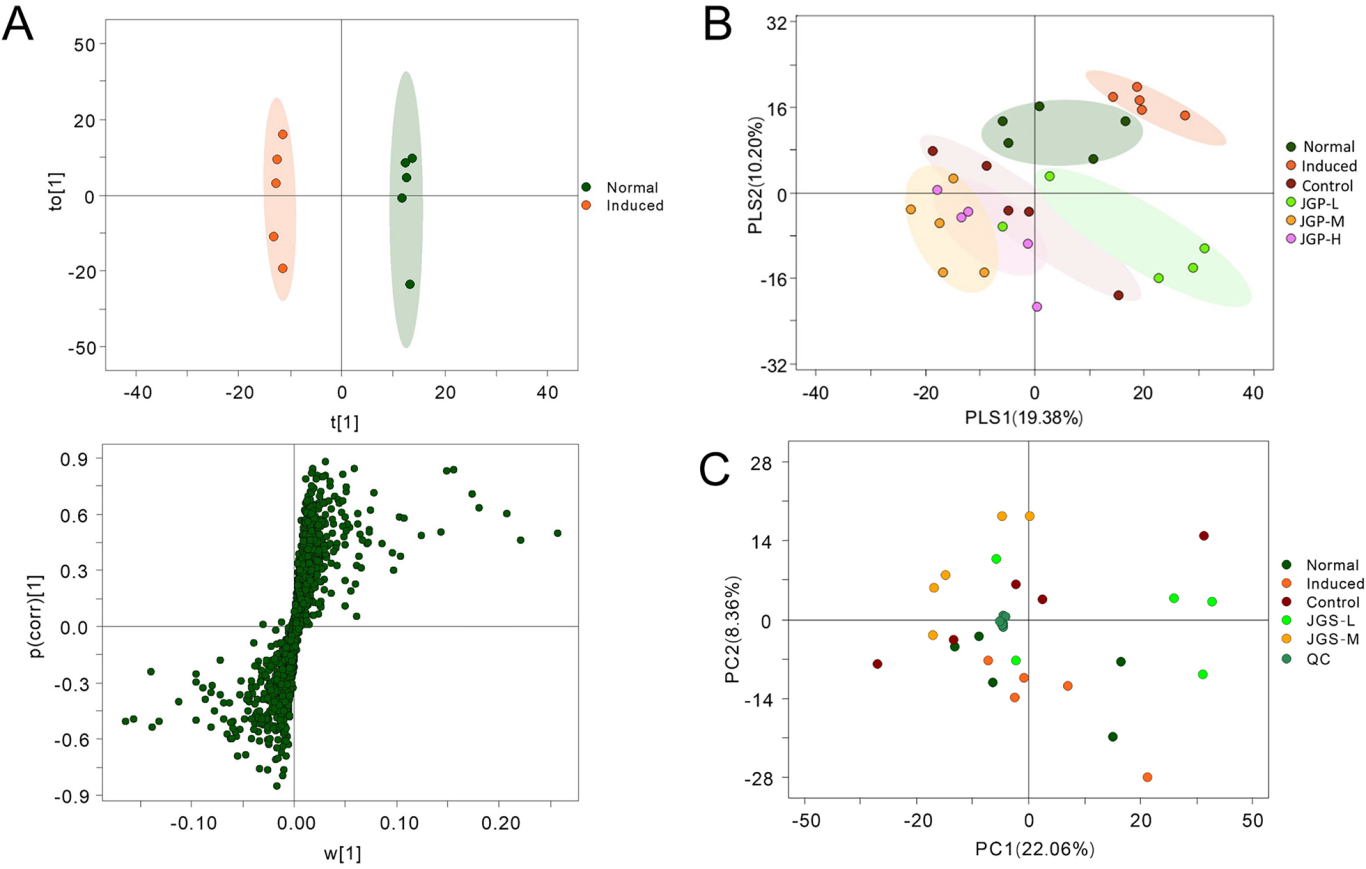
Multivariate analysis of metabolites in fecal samples from the experimental and control groups. **A** Principal component analysis (PCA) score plots and S-plots of orthogonal partial least squares discriminant analysis (OPLS-DA) models from the normal and model groups. **B** OPLS-DA of metabolic profiles in the experimental and control groups. **C** PCA of metabolic profiles in each group (five animals per group)

Alterations in gut microbial composition cause changes in the metabolic phenotype of the intestinal flora. Hence, metabolites in fecal samples across groups were identified. Differences across groups were assessed by OPLS-DA. The NG and MG clustered separately, indicating that metabolites in these groups were distinct (Fig. 6A). OPLS-DA and PCA showed that the PCG and JGP-treated groups clustered separately from the NG (Fig. 6B, C). JGP-treated groups clustered with the PCG, suggesting that JGP treatment changed metabolic profiles.

Differential metabolites were identified based on p-values and log_2_ fold-change. The number of metabolites differentially expressed in the NG vs. MG, MG vs. JGP-L, MG vs. JGP-M, and MG vs. JGP-H was 69, 133, 249, and 226, respectively (Additional file 1: Fig. S5A–C). BCG + LPS changed the levels of seven fecal metabolites associated with ILI (allylestrenol [AT], eplerenone, phosphatidylethanolamine (PE) (P-20:0/0:0), sphingomyelin (SM) d27:1, soyasapogenol C, chrysin, and soyasaponin I), and JGP treatment reversed this effect **(**Table 1**)**.

**Table 1:** The identified and change trend of the potential biomarkers of ILI mice intervened by JGP

No. VIP	Metabolites	Induced	JGP-L	JGP-M	JGP-H
11.99	Allylestrenol	^**#**^	ns	ns	^↑^**
21.94	Eplerenone	^**↓#**^	^↑^**	^↓^*	ns
31.37	PE (P-20:0/0:0)	^**↑#**^	ns	^↓^*	ns
41.78	SM d27:1	^**↓#**^	^**↑**^	ns	ns
51.84	Soyasapogenol C	^**↓#**^	^↑^*	ns	ns
62.17	Chrysin	^**↑#**^	ns	^↓^*	ns
71.66	Soyasaponin I	^**↓##**^	ns	^↑^***	ns

^**#**^ indicates a significant change between the normal and induced groups (^**#**^*P* < 0.05, ^**##**^*P* < 0.01); ∗ indicates significant change of different treatment groups vs Induced group (^*^P < 0.05; *P* < 0.01; ^***^P < 0.001). “ns” represents not significant

**Fig. 7:**
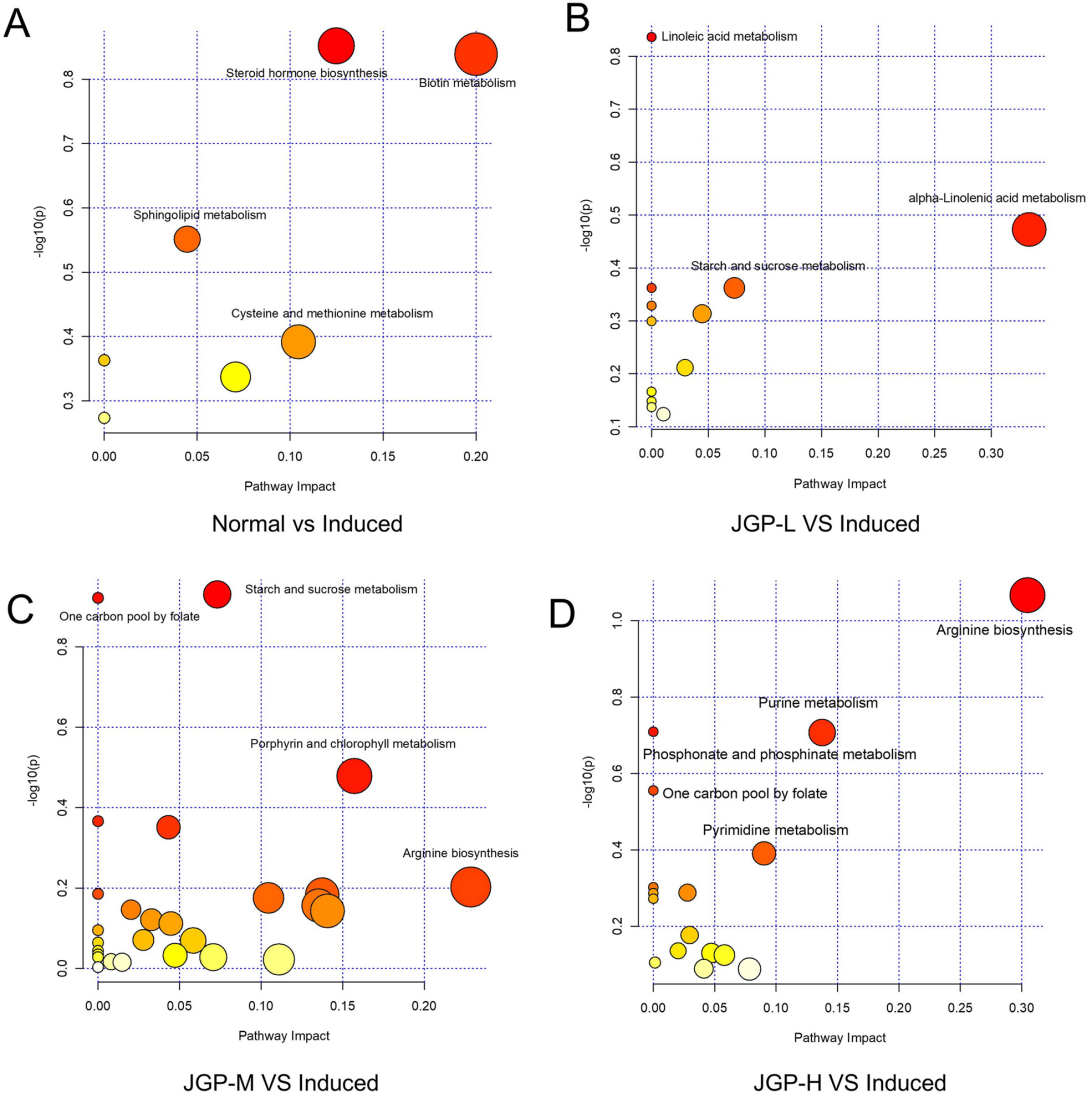
Enriched pathway analysis between groups. **A** Normal and model groups. **B** JGP-L and model groups. **C** JGP-M and model groups. **D** JGP-H and model groups

Pathway enrichment analysis was performed using MetaboAnalyst 5.0 (*p* < 0.05, impact value > 0.1). BCG + LPS significantly enriched biotin metabolism and steroid hormone biosynthesis **(**Fig. 7A–D**)**. Enriched metabolic pathways changed depending on the JGP dose. For instance, JGP-L stimulated alpha-linolenic acid metabolism, while JGP-M enriched starch and sucrose metabolism, porphyrin and chlorophyll metabolism, and arginine biosynthesis. In turn, JGP-H stimulated purine and pyrimidine metabolism and arginine biosynthesis. These findings indicate that JGP could potentially regulate the levels of metabolites and related metabolic pathways, modulating the development of ILI.

### Correlation between the gut microbiota, metabolic profile, and ILI indexes

**Fig. 8:**
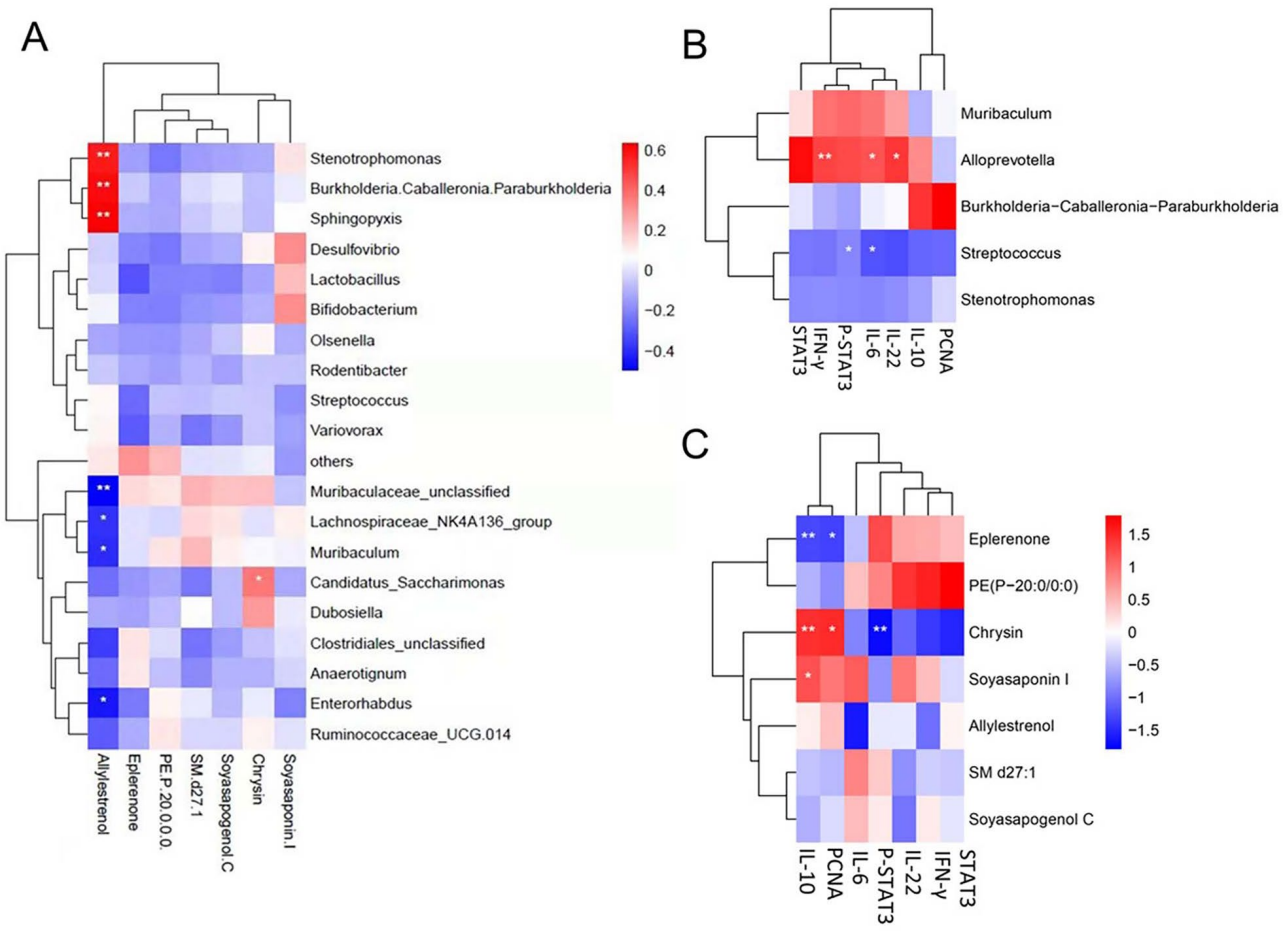
Pearson correlations analysis between gut microbiota, metabolites, and immunological liver injury (ILI) indexes. **A** Correlation between fecal metabolites and bacterial genera. **B** Correlation between ILI indexes and bacterial genera. **C** Correlation between feces metabolites and ILI indexes. ^*^|r|> 0.5 and *p* < 0.05, ^**^|r|> 0.5 and *p* < 0.01

Pearson correlations between bacterial genera and metabolites were determined. Allylestrenol, whose expression decreased in the MG, was positively correlated with *Stenotrophomonas*, *Burkholderia-Caballeronia-Paraburkholderia*, and *Sphingopyxis* and negatively associated with Muribaculaceae unclassified, Lachnospiraceae_NK4A136_group, *Muribaculum*, and *Enterorhabdus* (Fig. 8A)*.* Chrysin was strongly correlated with *Candidatus saccharimonas. Alloprevotella* was positively linked with IFN-γ, IL-6, and IL-22. Conversely, *Streptococcus* was negatively associated with p-STAT3 and IL-6 (Fig. 8B)). Eplerenone was negatively correlated with IL-10 and PCNA. Chrysin was positively associated with IL-10 and PCNA and negatively correlated with p-STAT3. Soyasaponin I was positively linked with IL-10 (Fig. 8C).

## Discussion

This study demonstrated that JGP improved ILI by restoring gut microbial composition and changing the metabolic profile. The impact of JGP on the gut microbiota was assessed by establishing a mouse model of ILI induced by BCG and LPS. We investigated the effects of JGP on the gut microbiome and the relationship between the microbiota and metabolic profiles in ILI mice by 16S rRNA gene sequencing and UPLC-MS-based metabolomics. The results suggest that JGP reduces ILI by restoring the level of *Alloprevotella*, *Burkholderia-Caballeronia-Paraburkholderia*, *Muribaculum*, *Streptococcus*, and *Stenotrophomonas*, and controlling the abnormality of biotin metabolism and steroid hormone production. Additionally, BCG + LPS changed the levels of AT, eplerenone, PE (P-20:0/0:0), SM d27:1, soyasapogenol C, chrysin, and soyasaponin I, whereas JGP treatment reversed this effect. This study elucidates the effects of JGP and demonstrates that the intestinal microbiota and associated metabolites are potential therapeutic targets for ILI.

Bacterial LPS induces fulminant hepatitis in BCG-treated mice [39, 40]. Inflammatory cells, including KCs, infiltrate the liver and synthesize and secrete inflammatory cytokines, excessive inflammatory cells, and pro-inflammatory and immunogenic factors, ultimately leading to endotoxin-induced liver injury [41, 42]. Animal models simulating ILI provide valuable insights into the pathophysiology of liver diseases.

KCs are the largest population of resident macrophages in the liver [43]. Activated KCs produce pro-inflammatory cytokines, including IL-6, IL-1, and tumor necrosis factor-alpha (TNF-α) [44, 45]. The inhibition of IFN-γ, IL-6, and IL-22 protects against BCG/LPS-induced ILI [46–49]. We found that JGP-H significantly decreased the percentage of KCs in ILI mice, potentially reducing liver damage induced by BCG + LPS. In addition, BCG + LPS significantly increased the protein expression of IFN-γ, IL-6, IL-10, and IL-22 in the liver, and JGP reduced the effect on the expression of IFN-γ, IL-6, and IL-22. These findings suggest that JGP has anti-inflammatory effects.

Hepatic STAT3 is upregulated in liver injury and is essential for liver cell apoptosis [50, 51]. BCG + LPS increased the protein expression of STAT3 and p-STAT3, suggesting that ILI is associated with liver cell apoptosis. Consequently, JGP decreased p-STAT3 expression, indicating that JGP reduces cell apoptosis by inhibiting the STAT3 signaling pathway.

The gut-liver axis integrates metabolites with the gut and liver [52]. Changes in intestinal flora are associated with liver diseases [16, 53, 54]. Previous studies have shown that the intestinal-liver axis is the physiological basis of the interaction between intestinal flora and liver. Under physiological conditions, the liver transports nutrients to the intestinal tract. The intestinal mucosal barrier ensures that the intestines absorb nutrients while limiting pathogens and microbial-derived molecules. However, under the condition of liver injury, the intestinal mucosal barrier function is destroyed, and the intestinal flora and their metabolites can reach the liver as antigen signals to induce inflammation and immune regulation. Using a multi-omics approach, we examined the influence of JGP on gut microbiota in ILI mice and the relationship between the gut microbiome and host metabolism. Bacterial 16S rRNA sequencing demonstrated that ILI increased gut microbial diversity, whereas JGP treatment considerably improved gut microbial diversity and richness. However, JGP-H treatment decreased microbial diversity and richness. PCoA revealed substantial differences in gut microbial structure between the NG and MG. Nevertheless, microbial structure was similar between the JGP-M, JGP-H, and NG, demonstrating that chemically-induced ILI altered microbial structure while JGP reversed this change. These results indicate that JGP treatment can maintain intestinal immune homeostasis associated with alterations of the intestinal flora.

JGP treatment restored the levels of *Alloprevotella*, *Burkholderia-Caballeronia-Paraburkholderia*, *Muribaculum*, *Streptococcus*, and *Stenotrophomonas* in ILI mice. *Alloprevotella*, from the phylum Bacteroidetes, has anti-inflammatory effects [55]. BCG + LPS increased the relative abundance of *Alloprevotella*, whereas JGP treatment reversed this effect. This result may be due to negative feedback regulation. Nonetheless, this feedback mechanism is unclear. *Muribaculum* may also be linked to inflammatory processes [56, 57]. These five bacterial genera are thought to mediate the effect of JGP.

Changes in intestinal flora metabolism in ILI mice were regulated by JGP treatment. Pathway enrichment analysis showed that JGP stimulated biotin metabolism and steroid hormone biosynthesis. JGP-M and JGP-H stimulated arginine biosynthesis. Arginine supplementation is known to reduce liver injury[58, 59]. The serum levels of arginine decrease in patients with acute liver injury [60, 61]. Therefore, blood arginine is a good indicator of liver regeneration after acute liver damage [60–62]. These findings suggest that arginine biosynthesis mediates the hepatoprotective effect of JGP.

BCG + LPS changed the levels of AT, eplerenone, PE (P-20:0/0:0), SM d27:1, soyasapogenol C, chrysin, and soyasaponin I, while JGP treatment reversed these effects. Notably, increased allylestrenol was significantly associated with changes in the relative abundance of several bacterial genera, and chrysin was positively correlated with *Candidatus saccharimonas*. AT is used clinically to treat abortion, intrauterine growth retardation, and prostatic hypertrophy [63, 64]. Moreover, AT combined with ritodrine significantly reduced the expression of inflammatory cytokines, including IL-6 and IL-10 [65]. However, we found no correlation between AT and ILI indexes, which might be because AT regulates the expression of other inflammatory factors.

Chrysin has anti-inflammatory and hepatoprotective effects [66, 67]. Correlation analysis showed that chrysin was positively linked with IL-10, and PCNA expression and negatively linked with p-STAT3. Soyasaponin I was positively correlated with IL-10, consistent with the anti-inflammatory properties of this compound [68, 69]. Therefore, AT, soyasaponin I, chrysin, and the gut microbiota may mediate the effects of JGP on ILI.

## Conclusion

JGP significantly reduced ILI by restoring gut microbial composition and stimulating metabolic pathways. These findings not only elucidate the therapeutic impact of JGP on ILI but also underscore its potential clinical utility.

### Supplementary Information

**Additional file 1: Fig. S1.** **A** Representative images of STAT3 staining (× 200). (**A** normal group; **B** model group; **C** positive control group; **D** JGP-L group; E: JGP-M group; F: JGP-H group). STAT3-positive cells are indicated by arrowheads. Bars = 100μm. **B** Percentage of STAT3-positive cells. Data were analyzed by one-way analysis of variance and were presented as mean ± SEM. ^#^*p* < 0.05, ^##^*p* < 0.01; ^###^*p* < 0.001. *p < 0.05; **p < 0.01; ***p < 0.001. **Fig. S2.** Flow cytometry analysis of the percentage of Kupffer cells (CD45^+^ CD11b^+^ F4/80^+^) and Ki67^+^ cells in the liver of control mice, model mice (induced for immunological liver injury), and model animals treated with Jian Gan powder. Data were analyzed by one-way analysis of variance and were expressed as mean ± SEM. *p < 0.05, **p < 0.01, ***p < 0.001. **Fig. S3.** **A** Species richness in fecal samples from the experimental and control groups **A**: normal group; **B** model group (induced for immunological liver injury); **C** positive control group; **D** JGP-L group; **E** JGP-M group; **F** JGP-H group). **B** Number of OTUs in the experimental and control groups. **Fig. S4.** **A** Associations between intestinal microbial genera. **B** Spearman correlation coefficients between gut microbial genera. **Fig. S5.** Heatmap **A** and volcano plot **B** of differentially expressed fecal metabolites. **C** Number of upregulated and downregulated fecal metabolites between the normal group and model group (MG, induced for immunological liver injury), JGP-L and MG, JGP-M and MG, and JGP-H and MG.

**Additional file 2: Table S1-1.** Characterization of chemical constituents of JGP by UHPLC-Q-TOF–MS analysis (positive ion mode). **Table S1-2.** Characterization of chemical constituents of JGP by UHPLC-Q-TOF–MS analysis (negative ion mode).

**Additional file 3.** Original WB images of Fig. 2C.

## Data Availability

The 16S rRNA gene sequencing data were submitted to the NCBI Sequence Read Archive (http://www.ncbi.nlm.nih.gov/sra) under the accession number PRJNA911190.

## Abbreviations

ILI: Immunological liver injury
JGP: Jian Gan powder
BCG: Bacillus Calmette-Guérin
IFN-γ: Interferon-γ
IL1/6/10/22: Interleukin 1/6/10/22
p-STAT3: Phosphorylated transducer and activator of transcription-3
LPS: Lipopolysaccharide
PE: Phosphatidylethanolamine
SM: Sphingomyelin
TCM: Traditional Chinese medicine
UHPLC: Ultra-high performance liquid chromatography
MS: Mass spectrometry
BCA: Bicinchoninic acid
NG: Normal group
MG: Model group
PCG: Positive control group
ELISA: Enzyme-linked immunosorbent assay
PBS: Phosphate-buffered saline
TBST: Tris-buffered saline and Tween
Q-PCR: Quantitative real-time polymerase chain reaction
DADA2: Divisive Amplicon Denoising Algorithm2
QIIME2: Quantitative Insights Into Microbial Ecology 2
KEGG: Kyoto Encyclopedia of Genes and Genomes
HMDB: Human Metabolome Database
QC: Quality control
PCA: Principal component analysis
OPLS‐DA: Orthogonal partial least squares discriminant analysis
VIP: Variable importance in projection
HE: Hematoxylin–eosin
LDA: Linear discriminant analysis
KC: Kupffer cell
PCNA: Proliferating cell nuclear antigen
PCoA: Principal coordinate analysis
AT: Allylestrenol
TNF-α: Tumor necrosis factor-alpha
