# Supplementary material for: Jian Gan powder ameliorates immunological liver injury in mice by modulating the gut microbiota and metabolic profiles
Source: Eur J Med Res. 2024 Apr 20;29:240. doi: 10.1186/s40001-024-01827-2 (PMC11031866; doi:10.1186/s40001-024-01827-2)
Supplement: Supplementary file 1 — Additional file 1: Fig. S1. A Representative images of STAT3 staining (× 200). (A normal group; B model group; C positive control group; D JGP-L group; E: JGP-M group; F: JGP-H group). STAT3-positive cells are indicated by arrowheads. Bars = 100μm. B Percentage of STAT3-positive cells. Data were analyzed by one-way analysis of variance and were presented as mean ± SEM. #p < 0.05, ##p < 0.01; ###p < 0.001. *p < 0.05; **p < 0.01; ***p < 0.001. Fig. S2. Flow cytometry analysis of the percentage of Kupffer cells (CD45+ CD11b+ F4/80+) and Ki67+ cells in the liver of control mice, model mice (induced for immunological liver injury), and model animals treated with Jian Gan powder. Data were analyzed by one-way analysis of variance and were expressed as mean ± SEM. *p < 0.05, **p < 0.01, ***p < 0.001. Fig. S3. A Species richness in fecal samples from the experimental and control groups A: normal group; B model group (induced for immunological liver injury); C positive control group; D JGP-L group; E JGP-M group; F JGP-H group). B Number of OTUs in the experimental and control groups. Fig. S4. A Associations between intestinal microbial genera. B Spearman correlation coefficients between gut microbial genera. Fig. S5. Heatmap A and volcano plot B of differentially expressed fecal metabolites. C Number of upregulated and downregulated fecal metabolites between the normal group and model group (MG, induced for immunological liver injury), JGP-L and MG, JGP-M and MG, and JGP-H and MG. [file 40001_2024_1827_MOESM1_ESM.docx]

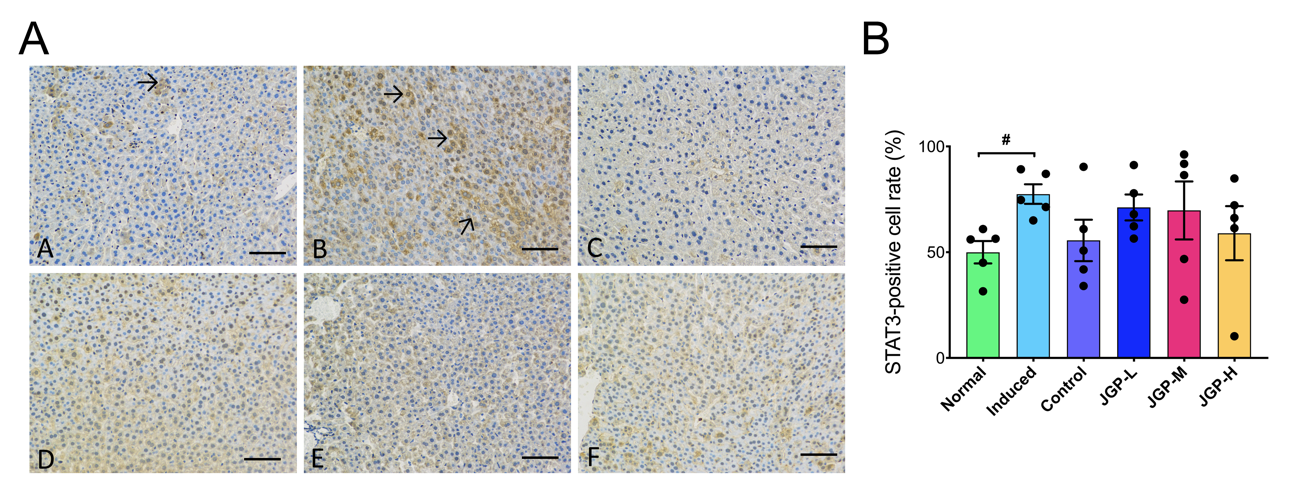


**Supplementary Figure 1.** (A) Representative images of STAT3 staining (×200). (A: normal group; B: model group; C: positive control group; D: JGP-L group; E: JGP-M group; F: JGP-H group). STAT3-positive cells are indicated by arrowheads. Bars=100 𝜇m. (B) Percentage of STAT3-positive cells. Data were analyzed by one-way analysis of variance and were presented as mean ± SEM. ^#^*p* < 0.05, ^##^*p* < 0.01; ^###^*p*< 0.001. **p* < 0.05; ***p* < 0.01; ****p* < 0.001.


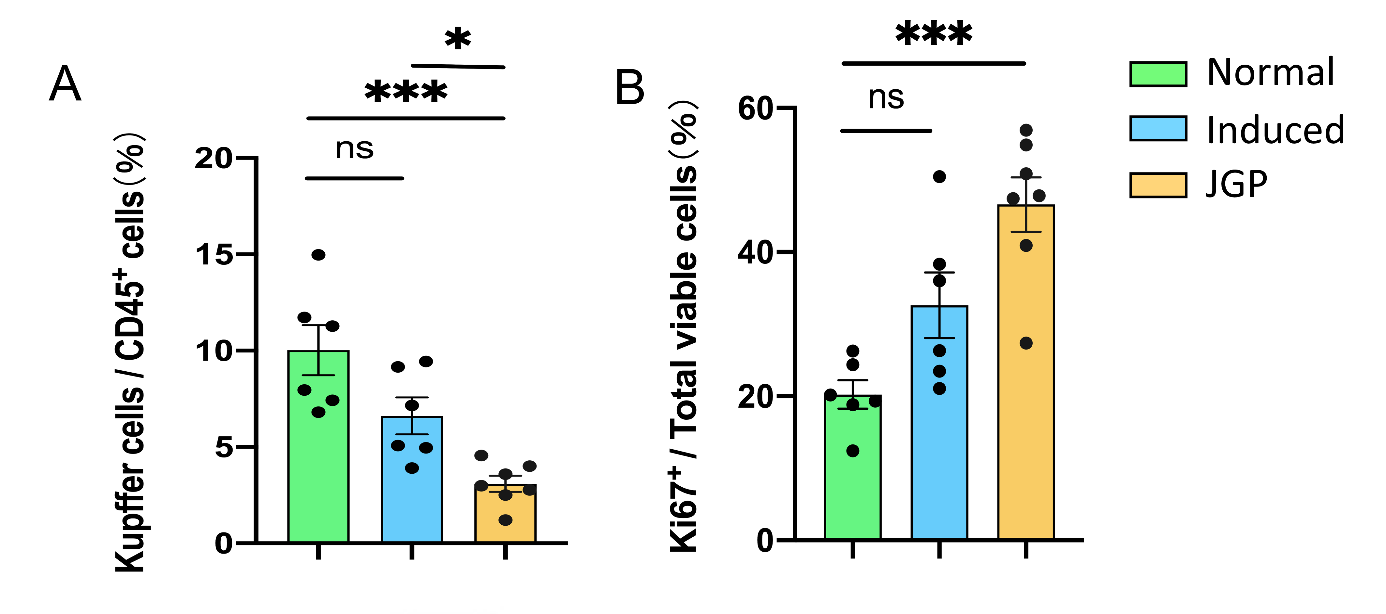


**Supplementary Figure 2.** Flow cytometry analysis of the percentage of Kupffer cells (CD45^+^ CD11b^+^ F4/80^+^) and Ki67^+^ cells in the liver of control mice, model mice (induced for immunological liver injury), and model animals treated with Jian Gan powder. Data were analyzed by one-way analysis of variance and were expressed as mean ± SEM. **p* < 0.05, ***p* < 0.01, ****p* < 0.001.


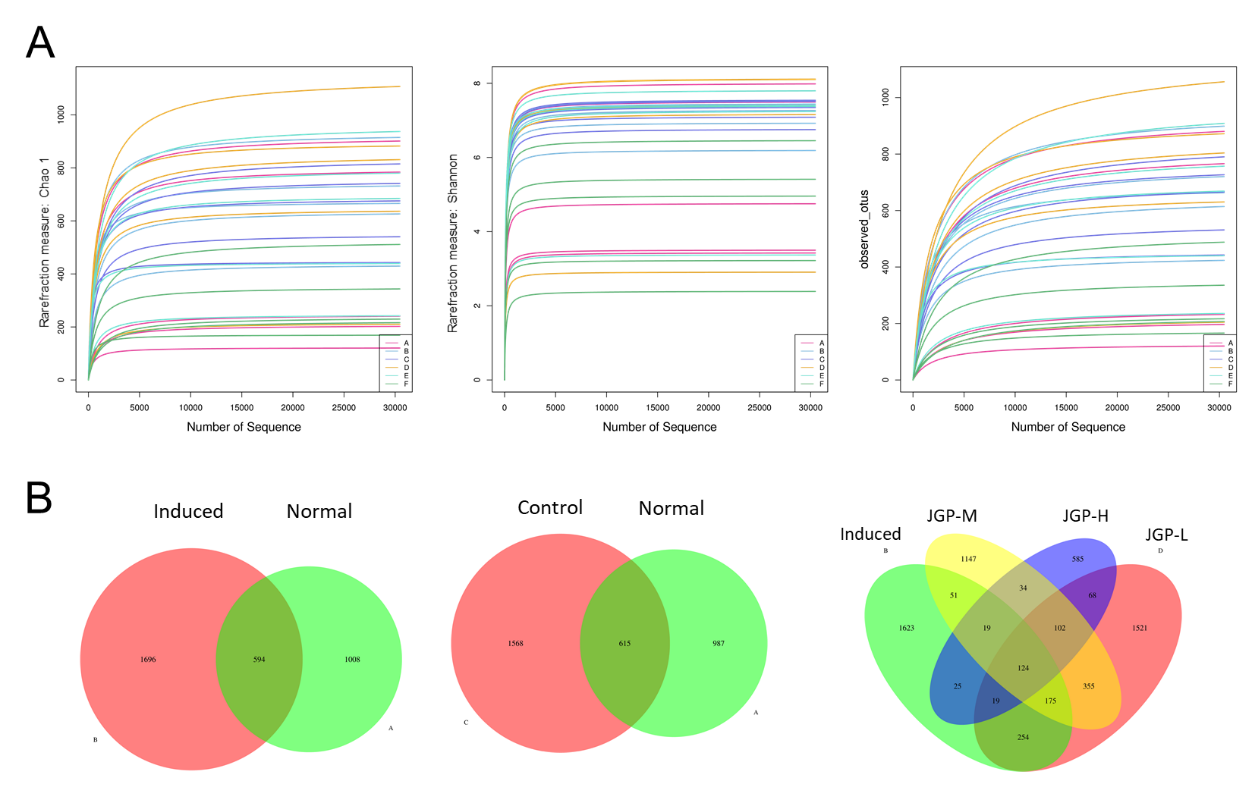


**Supplementary Figure 3.** (A) Species richness in fecal samples from the experimental and control groups (A: normal group; B: model group (induced for immunological liver injury); C: positive control group; D: JGP-L group; E: JGP-M group; F: JGP-H group). (B) Number of OTUs in the experimental and control groups.


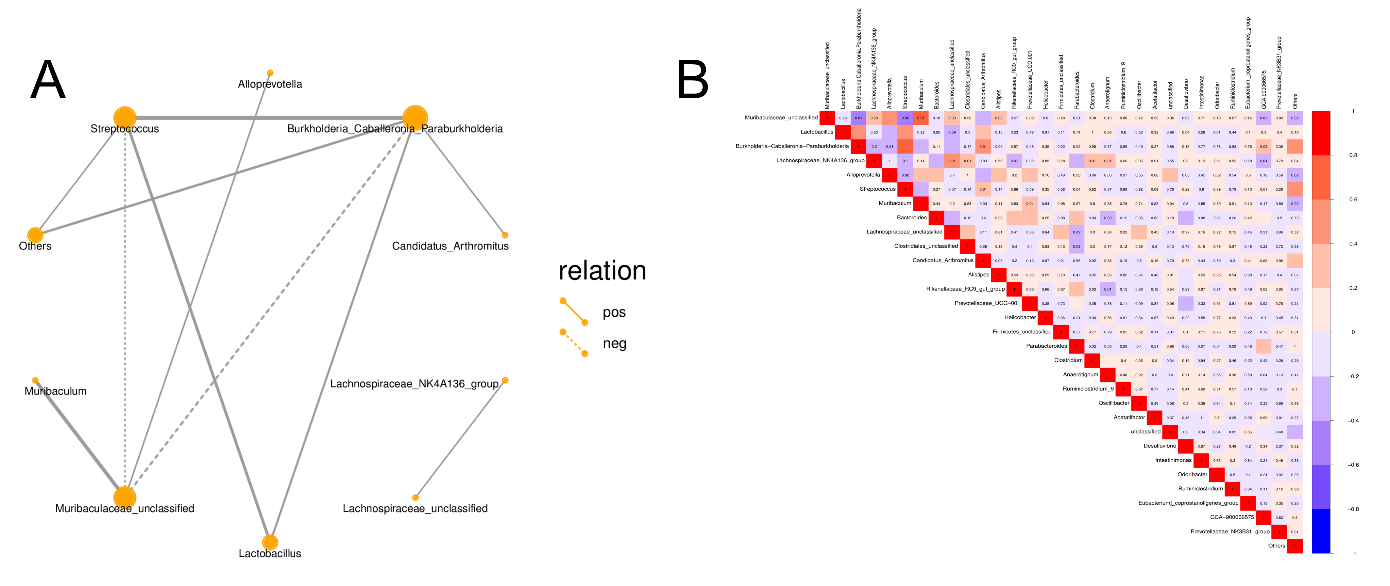


**Supplementary Figure 4.** (A) Associations between intestinal microbial genera. (B) Spearman correlation coefficients between gut microbial genera.

**
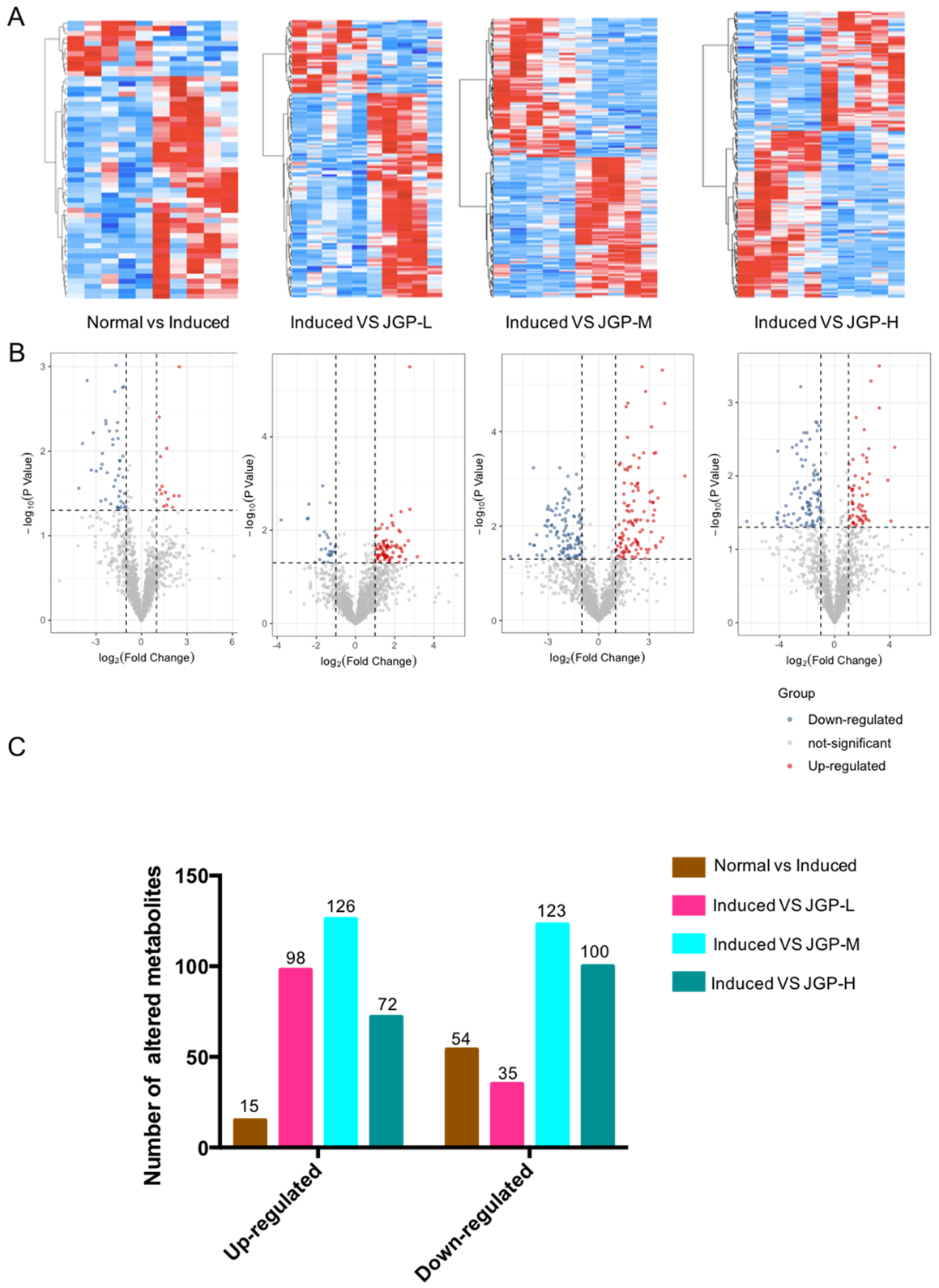
**

**Supplementary Figure 5.** Heatmap (A) and volcano plot (B) of differentially expressed fecal metabolites. (C) Number of upregulated and downregulated fecal metabolites between the normal group and model group (MG, induced for immunological liver injury), JGP-L and MG, JGP-M and MG, and JGP-H and MG.
