# Supplementary material for: Jian Gan powder ameliorates immunological liver injury in mice by modulating the gut microbiota and metabolic profiles
Source: Eur J Med Res. 2024 Apr 20;29:240. doi: 10.1186/s40001-024-01827-2 (PMC11031866; doi:10.1186/s40001-024-01827-2)
Supplement: Supplementary file 2 — Additional file 2: Table S1-1. Characterization of chemical constituents of JGP by UHPLC-Q-TOF–MS analysis (positive ion mode). Table S1-2. Characterization of chemical constituents of JGP by UHPLC-Q-TOF–MS analysis (negative ion mode). [file 40001_2024_1827_MOESM2_ESM.docx]

**Table S1-1** Characterization of chemical constituents of JGP by UHPLC-Q-TOF-MS analysis (positive ion mode)

| No. | tR(min) | Experimental mass (m/z) | Precursor ion | Identification | Formula | Source |
| --- | --- | --- | --- | --- | --- | --- |
| 1 | 0.954 | 381.0789, 543.1312 |  |  |  | G、R、S |
| 2 | 1.104 | 191.1176, 205.1334 |  |  |  |  |
| 3 | 1.382 | 132.1017 |  |  |  | G、R、AS、B、C、GR、SF |
|  |  | 265.1906 | [M+H]^+^ | oxymatrine, oxysophoridine | C_15_H_24_N_2_O_2_ | SF |
|  |  | 281.1854 |  |  |  | SF |
|  |  | 245.1642 | [M+H]^+^ | anagyrine, sophoramine | C_15_H_20_N_2_O | SF |
| 4 | 1.661 | 249.1967 | [M+H]^+^ | sophoridine, matrine | C_15_H_24_N_2_O | SF、R |
| 5 | 2.043 | 265.1913 | [M+H]^+^ | oxymatrine, oxysophoridine | C_15_H_24_N_2_O_2_ | SF、R |
| 6 | 2.867 | 265.1907 | [M+H]^+^ | oxymatrine, oxysophoridine | C_15_H_24_N_2_O_2_ | SF |
| 7 | 5.083 | 922.0081 |  |  |  |  |
| 8 | 5.561 | 481.1697 | [M+H]^+^ | alibiflorin | C_23_H_28_O_11_ | P |
| 9 | 7.22 | 273.0755, 419.1341, 581.1871 |  |  |  |  |
| 10 | 7.386 | 340.2597, 679.5105, 701.4922 |  |  |  |  |
| 11 | 8.147 | 340.2599, 431.1326, 521.1065, 679.5104, 922.0074 |  |  |  |  |
| 12 | 8.2424 | 480.2777, 498.2881 |  |  |  |  |
| 13 | 8.475 | 480.2772, 498.2881 |  |  |  |  |
| 14 | 9.494 | 423.3642 | [M+H-Glc-Glc-H_2_O]^+^ | Rg1、Rf、Re | C_42_H_72_O_14_ | G |

A stands for *Aurantii Fructus Immaturus*; AS stands for *Artemisiae Scopariae Herba*; B stands for *Bupleuri Radix*; C stands for *Cyperi Rhizoma*; G stands for *Ginseng Radix* et *Rhizoma*; GR stands for *Glycyrrhizae Radix* et *Rhizoma*; P stands for *Paeoniae Radix*; R stands for *Astragali Radix*; S stands for *Salviae Miltiorrhizae Radix* et *Rhizoma*; SF stands for *Sophorae Flavescentis Radix.*

**Table S1-2** Characterization of chemical constituents of JGP by UHPLC-Q-TOF-MS analysis (negative ion mode)

| No. | tR(min) | Experimental mass (m/z) | Precursor ion | Identification | Formula | Source |
| --- | --- | --- | --- | --- | --- | --- |
| 1 | 0.962 | 341.1105 | [M+COOH]^-^ | przewaquinone C, Danshenxinkun A | C_18_H_16_O_4_ | S |
|  |  |  |  | isocryptotanshinone, cryptotanshinone | C_19_H_20_O_3_ | S |
|  |  |  |  | saprorthoquinone, Aethiopinone | C_20_H_24_O_2_ | S |
|  |  | 503.1644 |  |  |  |  |
| 2 | 2.209 | 255.0521 |  |  |  | R、SF |
| 3 | 5.406 | 687.2164 | [M+COOH]^-^ | isomaltopaeoniflorin | C_29_H_38_O_16_ | P |
| 4 | 5.594 | 525.1641 | [M+COOH]^-^ | alibiflorin | C_23_H_28_O_11_ | P |
| 5 | 5.914 | 367.1061 |  |  |  | G |
|  |  | 525.1640 | [M+COOH]^-^ | paeoniflorin | C_23_H_28_O_11_ | P |
| 6 | 6.595 | 649.2538 |  |  |  | A |
|  |  | 549.1651 | [M-H]^-^ | isoliquiritin apioside, liquiritin apioside, isoglycyrrhizin glucose apigenin | C_26_H_30_O_13_ | GR |
| 7 | 7.232 | 537.1106 | [M-H]^-^ | Salvianolic acid H/I | C_27_H_22_O_12_ | S |
| 8 | 7.560 | 607.1702 | [M+COOH]^-^ | Kushenol O | C_27_H_30_O_13_ | SF |
|  |  | 609.1861 | [M-H]^-^ | hesperidin | C_28_H_34_O_15_ | A |
| 9 | 8.138 | 137.0248 | [M-H]^-^ | salicylic acid | C_7_H_6_O_3_ | AS |
|  |  | 717.1497 | [M-H]^-^ | Salvianolic acid B, Salvianolic acid E | C_36_H_30_O_16_ | S |
| 10 | 8.263 | 633.2573 |  |  |  | A |
| 11 | 8.496 | 514.2872 |  |  |  |  |
| 12 | 8.953 | 255.0681 | [M-H]^-^ | liquiritigenin, isoliquiritigenin | C_15_H_12_O_4_ | GR |
|  |  | 417.0828 | [M-H]^-^ | Natsudaidain | C_21_H_22_O_9_ | A |
| 13 | 9.522 | 845.4986 | [M+COOH]^-^ | Rg1, Rf, Rg7 | C_42_H_72_O_14_ | G |
| 14 | 10.117 | 271.0627 | [M-H]^-^ | naringenin | C_15_H_12_O_5_ | A |
| 15 | 10.890 | 821.4014 | [M-H]^-^ | glycyrrhizic acid, Uralum saponin B | C_42_H_62_O_16_ | GR |

A stands for *Aurantii Fructus Immaturus*; AS stands for *Artemisiae Scopariae Herba*; B stands for *Bupleuri Radix*; C stands for *Cyperi Rhizoma*; G stands for *Ginseng Radix* et *Rhizoma*; GR stands for *Glycyrrhizae Radix* et *Rhizoma*; P stands for *Paeoniae Radix*; R stands for *Astragali Radix*; S stands for *Salviae Miltiorrhizae Radix* et *Rhizoma*; SF stands for *Sophorae Flavescenti*
