# Supplementary material for: Jian Gan powder ameliorates immunological liver injury in mice by modulating the gut microbiota and metabolic profiles
Source: Eur J Med Res. 2024 Apr 20;29:240. doi: 10.1186/s40001-024-01827-2 (PMC11031866; doi:10.1186/s40001-024-01827-2)
Supplement: Supplementary file 3 — Additional file 3. Original WB images of Fig. 2C. [file 40001_2024_1827_MOESM3_ESM.docx]

**Supplementary 3.** Original WB images of Figure 2C.
